# The psychological processes involved in patient empowerment

**DOI:** 10.1186/1750-1172-7-S2-A31

**Published:** 2012-11-22

**Authors:** Isabelle Aujoulat, Bridget Young, Peter Salmon

**Affiliations:** 1 Institute of Health & Society, Université Catholique de Louvain, Bruxelles, Belgium; 2 Division of Clinical Psychology, University of Liverpool, Liverpool, UK

## 

Patient empowerment is widely accepted as a process by which people are helped to use autonomous decision making in order to better self-manage their condition, gain control over their health and remain socially integrated [1]. Prevalent definitions of empowerment are therefore mainly based on the view that autonomy is recognised in people’s self-determination, and that empowerment is a procedural process of giving (provider to patient) and taking (patient from provider) power. Such definitions tend to minimise the uncontrollability which is inherent to living with severe health conditions. In such contexts, autonomy may be alternatively regarded as feeling secure in caring relationships [2], and having ‘ownership’ for one’s decision might be just as appropriate and empowering as participating in ‘shared decision-making’ [3]. This points the need to consider other approaches to patient empowerment. Living with a chronic health condition, particularly if the condition is progressive and disabling, implies that patients and families embrace three equally important challenges: managing illness work, managing everyday tasks, and coping with the need to maintain (or reconstruct) a continuous and valuable sense of self [4]. The third challenge is not sufficiently addressed in prevalent definitions of empowerment. Patient empowerment needs to be seen as a dynamic and creative process that is shaped by the individual’s own activity and yet acknowledges the individual’s dependence on others. As such, it is closer to the logic of care than the logic of choice [5]. In the logic of care, patient empowerment depends on not only the need to develop a sense of choice and control, but also the need to: i) feel secure and connected and; ii) develop a sense of meaning and coherence [6,7]. Patients’ need for competence and control is generally well addressed in self-management support interventions that promote cognitive and behavioural efforts to enhance self-efficacy and performance. By contrast, patients’ need for coherence and meaningfulness, which involves relinquishing control when challenges are perceived as being uncontrollable, is insufficiently acknowledged. *Taking and relinquishing control* should be seen as two interdependent aspects of a single dynamic process, rather than two opposites on a continuum from disempowerment to empowerment. As far as the need for security and connectedness is concerned, recent theoretical developments point to the importance of looking at how people’s attachment styles, shaped through previous experience of dependent relationships, might influence the patient-provider relationship [8]. Research is needed to understand how adaptive interventions may be built into health-care systems to more comprehensively support the complex psychological processes that underpin patient empowerment.

## References

[B1] The Lancet Patient empowerment-Who empowers whom? The Lancet 2012 379 1677 10.1016/S0140-6736(12)60699-0 22559881

[B2] Kukla R Conscientious Autonomy: Displacing Decisions in Health Care Hastings Cent Rep 2005 35 34 44 15957317

[B3] Mendick N Young B Holcombe C Salmon P The ethics of responsibility and ownership in decision-making about treatment for breast cancer: triangulation of consultation with patient and surgeon perspectives Soc Sci Med 2010 70 1904 1911 10.1016/j.socscimed.2009.12.039 20382463

[B4] Corbin J Strauss A Accompaniments of Chronic Illness: Changes in Body, Self, Biography, and Biographical Time Res Sociol Health Care 1987 6 249 281

[B5] Mol A The Logic of Care: Health and the Problem of Patient Choice 2008 Oxford, Routledge Press

[B6] Aujoulat I Luminet O Deccache A The perspective of patients on their experience of powerlessness Qualitative Health Research 2007 17 772 785 10.1177/1049732307302665 17582020

[B7] Aujoulat I Marcolongo R Bonadiman L Deccache A Reconsidering patient empowerment in chronic illness: A critique of models of bodily control and self-efficacy Soc Sci Med 2008 66 5 1228 1239 10.1016/j.socscimed.2007.11.034 18155338

[B8] Salmon P Young B Dependence and caring in clinical communication: the relevance of attachment and other theories Patient Educ Couns 2009 74 331 338 10.1016/j.pec.2008.12.011 19157761 PMC3764431

